# Effect of serum progesterone on human chorionic gonadotropin trigger day / metaphase II oocyte ratio on pregnancy and neonatal outcomes in women undergoing ICSI cycle

**DOI:** 10.1186/s12884-023-05549-x

**Published:** 2023-04-04

**Authors:** Li-Juan Huang, Qi Wan, Tian Li, Xing-Yu Lv, Li-Hong Geng, Qi-Qi He, Zhao-Hui Zhong, Yuan Li, Xiao-Jun Tang

**Affiliations:** 1 Research Center for Medical and Social Development, School of Public Health, Chongqing Medical University, Chongqing, 400016 China; 2 Xinan Gynecological Hospital, Chengdu, 610011 China; 3 The Department of Reproductive Medicine, the First Affiliated Hospital of Chongqing Medical University, Chongqing, 400016 China

**Keywords:** Progesterone on human chorionic gonadotropin trigger day/metaphase II oocyte (P/MII) ratio, Cut-off value, Pregnancy outcomes, Neonatal outcomes, ICSI

## Abstract

**Background:**

The serum progesterone on human chorionic gonadotropin trigger day / metaphase II oocyte (P/MII) ratio might be a more predictable indicator of pregnancy and neonatal outcomes as compare to P/estradiol (E2) or P alone. Hence, we conducted a larger population study to compare the pregnancy and neonatal outcomes in the low and high P/MII ratio.

**Methods:**

A retrospective, single-center, larger population cohort study between January 2015 and August 2021. Calculate the threshold effect of P/MII ratio on clinical pregnancy rate according to the construct smooth curve fitting. Divide data into two groups by threshold for comparison.

**Results:**

3566 fresh ICSI-ET cycles were included, in which 929 singleton delivery and 676 twin deliveries. Compare to P/MII ≤ 0.367 group, it indicated that the P/MII > 0.367 group had a lower clinical pregnancy rate and live birth rate, furthermore, a significantly higher rate of LBW and SGA were observed in the singleton and twin deliveries. No deleterious impact of high P/MII ratio on embryo quality and undesirable pregnancy outcomes was shown.

**Conclusions:**

When P/MII is higher than 0.367, may have adverse impacts on pregnancy and neonatal outcomes for ICSI cycle.

## Background

Use of exogenous gonadotropins for development of multiple dominant follicles during controlled ovarian stimulation (COS) [1], may result in serum progesterone (P) and estradiol (E2) supraphysiological elevation in the late follicular phase and on human chorionic gonadotropin (HCG) trigger day in in vitro fertilization (IVF) treatment. The supraphysiologic hyperprogesterone and hyperestrogenic milieu may lead to some unfavorable pregnancy and perinatal outcomes, by imposing an adverse uterine environment for implantation [2, 3].

Therefore, it is meaningful to explore the effect of P and E2 changes on HCG trigger day on pregnancy outcomes. The negative effect of hyperestrogenic milieu on fetal low birth weight has been confirmed [4], however, meta-analysis concluded that there was no difference in clinical pregnancy rates between low and high E2 levels on the HCG trigger day [5]. Studies have found that elevated P on HCG trigger day was related to the decrease of clinical pregnancy rate and live birth rate in different ovarian responses [6–12], however a study showed that P had a negatively effect on live birth rate both at lower and higher values [13]. Recent study proposed E2 alone or P/E2 ratio as an indicator for pregnancy outcomes [14], however, P/E2 ratio has low predictive value [15–17].

Shuffaro et al. propose that elevated P may be only harmful if it represents an increased production of P per follicle [18]. As P level elevation may correlate with the number of hormonally active oocyte, the P/oocyte ratio reflects the average amount of P produced by each oocyte on the day of final oocyte maturation [18, 19]. Several studies have demonstrated that P/follicle or P/MII oocyte ratio are good indicator to predict pregnancy outcome, but these studies were limited by relatively small sample sizes [14, 19–22]. Another problem is that the current studies mainly evaluate the effect of P/MII ratio on clinical pregnancy, with little consideration for neonatal outcomes after ICSI.

To address above questions, we performed a large retrospective study to explore the relationship between P/MII ratio and pregnancy and neonatal outcomes on ICSI cycle in patients with normal ovarian response.

## Materials and methods

### Study population and design

We performed a retrospective, single-center cohort study of women aged 18–40 years undergoing fresh ICSI cycle from January 2015 to August 2021. Patients’ demographic characteristics and laboratory features of patients were obtained at the Xinan Gynecological Hospital in Sichuan, China. Patients were excluded from the study if they experienced one of the following conditions: (1) baseline day 3 FSH > 12 IU/L; (2) patients with polycystic ovary syndrome and hyperprolactinemia; (3) endometriosis, submucous myoma of uterus, endometrial polyps, uterine inflammation, uterine malformation or ant uterine pathology that could affect implantation; (4) Patients with poor ovarian response according to the Bologna criteria [23]; (5) Patients using frozen sperm; (6) patients with cancelling transplantation cycle or PGT-A cycles or missing data. In total, 3566 fresh ICIS-ET cycles were included in the study. The study was reviewed and approved by the Ethics Committee of Chongqing Medical University.

### Controlled ovarian stimulation protocols

The COS protocols were chosen accorded to patients’ characteristics and physicians’ judgment. COS was performed using four following protocols. The luteal phase short-acting GnRH agonist long protocol: From the mid-luteal phase of the menstrual cycle, 0.1 mg short-acting GnRH agonist (Triptorelin Acetate, Ferring GmbH, Germany) was injected subcutaneously for 14–16 days until pituitary down-regulation was confirmed (follicle diameter ≤ 5 mm, E2 < 50 pg/ml, LH < 5 mIU/ml). Then the gonadotropin (Gn) (recombinant follicle-stimulating hormone (FSH), Gonalfin, Merck Serono, Switzerland) with an initial dose of 100–225 IU/d was administered until the follicles matured. The luteal phase long-acting GnRH agonist long protocol: From the mid-luteal phase of the menstrual cycle, a 3.75 mg long-acting GnRH agonist was injected subcutaneously, and pituitary down-regulation (follicle diameter ≤ 5 mm, E2 < 50 pg/ml, LH < 5 mIU/ml) was confirmed 14–16 days after injection. Then the gonadotropin (Gn) (recombinant FSH, Gonalfin, Merck Serono, Switzerland) with an initial dose of 225 IU/d was administered until the follicles matured. The GnRH antagonist protocol: Gn (recombinant FSH, Gonalfin, Merck Serono, Switzerland) generally 100–300 IU/d was administered on the 2nd to 4th day of menstruation. When the leading follicle reached 12 mm or when the Gn was used to 6–7 days, a 0.25 mg of GnRH antagonist (Cetrotide, Merck Serono S.p.A., Rome, Italy; Orgalutran, Organon, MSD-Italy) was injected subcutaneously daily until the ovulation trigger day. The follicular phase GnRH protocol: On the 2ed to 5th day of menstrual cycle and a 3.75 mg of GnRH agonist (Dafiline, Beaufour Ipsen, France) was injected subcutaneously. Down-regulation (follicle diameter ≤ 5 mm, estradiol (E2) < 50 pg/ml, luteinizing hormone (LH) < 5 mIU/ml) was confirmed after 28–37 days.

### Embryo culture and grading

Ovulation was triggered by HCG (Ovitrelle; Merck Serono S.p.A.) when the dominant follicles reached a diameter of 18 mm was observed by ultrasound. Oocyte retrieval was carried out 36 h after HCG administration by transvaginal ultrasound-guided puncture of follicles. The number of MII was counted 2–3 h after oocyte retrieval. The criteria to perform ICSI in our hospital were as followed: (1) severe oligozoospermia, asthenozoospermia and teratospermia, (2) obstructive azoospermia, (3) fertilization failure or fertilization rate < 25% in previous cycles, (4) abnormal acrosome function of sperm, (5) in vitro maturation (IVM) for oocytes, (6) preimplantation genetic testing (PGT). The cleavage stage embryos quality was evaluated according to the number of blastomeres, the rate of fragmentation and cell size uniformity, and the embryos with grade 1,2 were regarded as good-quality embryos [24]. The blastocyst quality was evaluated according to the expansion stage, inner cell mass and trophoblast cells, and 5AA, 5BB, 4AB, 4BA were regarded as good-quality blastocysts [25, 26]. ET following ICSI are typically performed at the cleavage-stage or blastocyst-stage transfer. The luteal phase was supported by Dydrogesterone (10 mg once, three times a day, Abbott Biologicals B.V., netherlands) combined with Intravaginal CrInone Progesterone (90 mg once a day, Merck Serono S.p.A., Rome, Italy).

### Hormone measurements

Serum P was measured on HCG trigger day using chemiluminescence immunoassay (Beckman-Coulter Unicel DxI 800 Access Analyzer;Beckman-Coulter, USA). The sensitivity of the P assay was 0.10ng/ml, the intra-assay and inter-assay coefficients of variation were 8.18% and 7.89%. All serum P measurements were performed in the same laboratory and using the same analytical method. Immunoassay System periodically calibrated to minimize changes in results associated with time and reagent batch updates.

### Definition of outcomes and statistical analysis

The implantation rate was defined by the ratio of the number of gestational sacs to the number of transferred embryos. Clinical pregnancy was defined by the observation of embryo sac and fetal heartbeat 28 days after ET by ultrasound. pregnancy loss was defined as a loss of clinical pregnancy before the 24th gestational week, ectopic pregnancy was defined as at least one gestational sac outside the uterine cavity. Telephone follow-up until delivery, and live birth was defined as the delivery of live infants at or after 28 weeks of pregnancy. Preterm birth (PTB) was defined as delivery before 37 completed weeks of gestation. Low birth weight (LBW) and macrosomia refer to a birth weight < 2500 g and > 4000 g. Small for gestational age (SGA) and large for gestational age (LGA) refer to a birth weight below the 10th percentile and greater than the 90th percentile for the gestational age at birth, respectively.

The SPSS version 26.0 and R version 4.2.2 were used to all analysis. Calculate the threshold effect of P/MII on clinical pregnancy rate according to the construct smooth curve fitting. Divide data into two groups according to threshold. Kolmogorov-Smirnov tests was used to check for normality. Mean ± Standard deviation was expressed for continuous variables and the Independent-Samples T test was used for comparison between the two groups. Percentages was expressed for categorical variables and the Pearson chi-square test or Fisher’s exact test was used for comparison between the two groups. Multivariate logistic regression models were performed to adjust for potential confounders and to calculate the odds ratios (OR) and the 95% confidence intervals (CI). A value of P < 0.05 was considered as significant.

## Result

### Association between P/MII ratio and clinical pregnancy

**Fig. 1:**
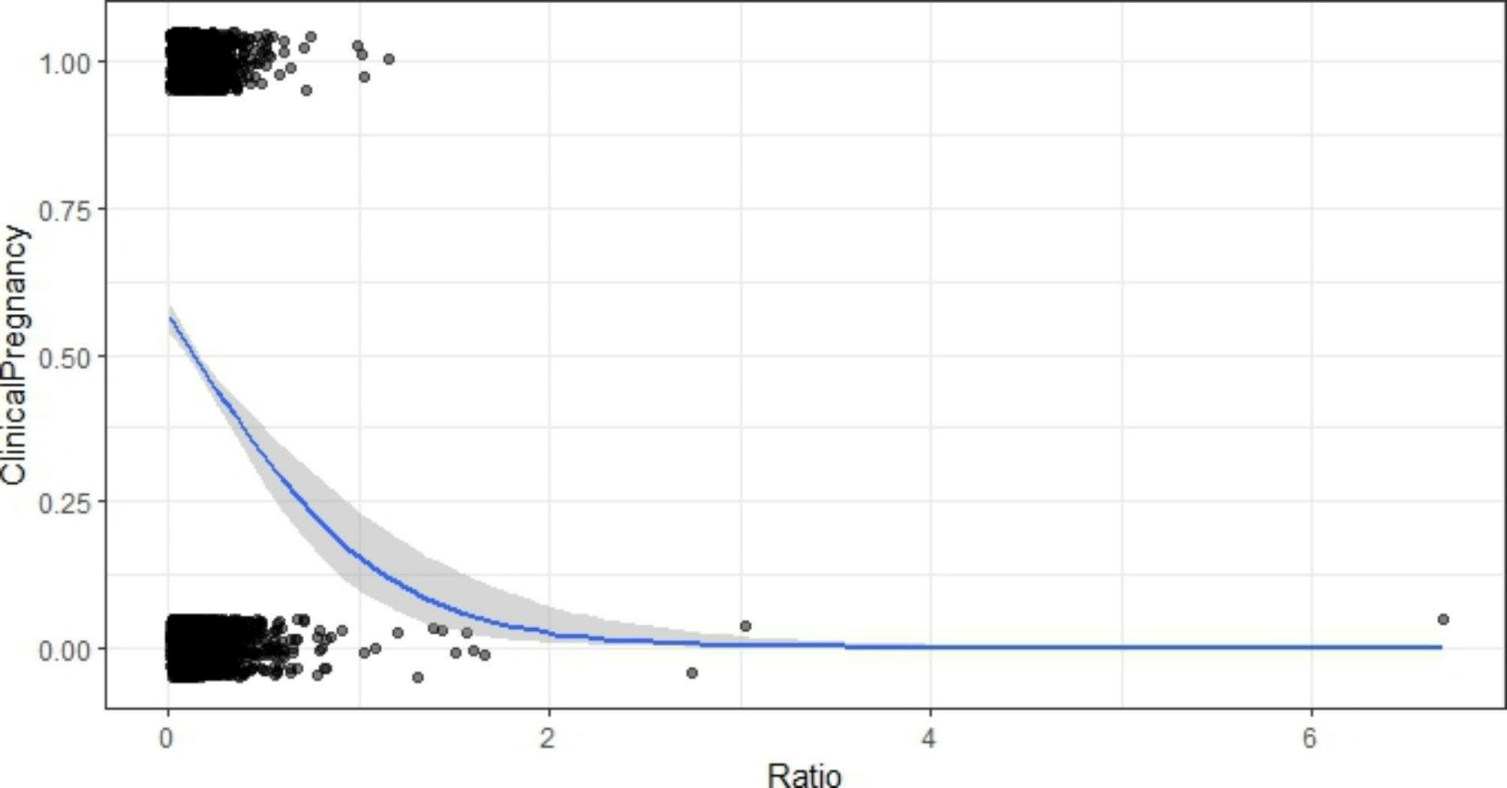
Curve fitting model of P/MII ratio and clinical pregnancy The threshold effect analysis shows that the P/MII inflection point is 0.367. When P/MII > 0.367, the clinical pregnancy rates decreased sharply with the increase in P/MII ratio (OR = 0.23, 95% CI 0.13–0.32, P < 0.001). When P/MII ≤ 0.367, no statistical significance in clinical pregnancy rate and P/MII ratio (P > 0.05)

A piecewise linear regression relationship between P/MII ratio and clinical pregnancy was observed (Fig. 1), and two-piecewise linear regression model was constructed to calculate the threshold effect of P/MII ratio on clinical pregnancy. The threshold effect analysis shows that the P/MII inflection point is 0.367. When P/MII > 0.367, the clinical pregnancy rates decreased sharply with the increase in P/MII ratio (OR = 0.23, 95% CI 0.13–0.32, P < 0.001). When P/MII ≤ 0.367, the relationship between P/MII ratio and clinical pregnancy rate was not statistically significant.

### Baseline characteristics

A total of 3566 fresh ICSI-ET cycles were included in the study, in which 929 singleton delivery and 676 twin deliveries. Participants were divided into two groups according to the threshold value: group 1 ≤ 0.367 (n = 3387), group 2 > 0.367 (n = 179). Baseline characteristics between two groups showed statistically significant differences in patients age, COS protocols, AFC, total dose of Gn, P level on HCG trigger day, E2 level on HCG trigger day (pg/mL), the number of oocytes retrieved, the number of MII oocytes, the number of embryo transferred (Table 1).

**Table 1:** Baseline characteristic in the fresh ICSI cycles

	group 1 ≤ 0.367(n = 3387)	group 2 > 0.367(n = 179)	P
Age (years)	30.45 ± 4.06	32.25 ± 4.05	< 0.001
BMI (kg/m^2^)	22.22 ± 2.90	22.35 ± 2.62	0.540
Infertility type			
Primary	2091 (61.7%)	118 (65.9%)	0.270
Secondary	1296 (38.3%)	61 (34.1%)	
Infertility duration(years)	4.13 ± 2.96	4.29 ± 3.23	0.483
COS protocols			
luteal phase short-acting GnRH agonist long protocol	929 (27.4%)	53 (29.6%)	< 0.001
luteal phase long-acting GnRH agonist long protocol	408 (12.0%)	33 (18.4%)	
GnRH antagonist protocol	932 (27.5%)	62 (34.6%)	
Follicular phase GnRH protocol	1118 (33.0%)	31 (17.3%)	
Basal FSH	7.19 ± 1.84	7.33 ± 1.75	0.302
Basal E2	52.25 ± 36.90	55.42 ± 31.07	0.259
Basal P	0.95 ± 2.13	1.24 ± 2.63	0.076
Basal LH	4.33 ± 2.76	4.30 ± 2.14	0.877
AFC	14.34 ± 6.41	11.42 ± 5.83	< 0.001
dose of Gn (U)	2004.98 ± 635.29	2220.76 ± 622.20	< 0.001
Gn duration (days)	10.41 ± 1.98	10.22 ± 2.20	0.207
P level on hCG day (ng/mL)	0.93 ± 0.46	1.37 ± 0.66	< 0.001
E2 level on hCG day (pg/ml)	2720.07 ± 1498.18	2008.62 ± 1282.35	< 0.001
Endometrial thickness on hCG day	10.69 ± 1.86	10.65 ± 2.12	0.811
Day of embryo transfer			
Day 3	2886 (85.2%)	162 (90.5%)	0.050
Day 5	501 (14.8%)	17 (9.5%)	
No. of retrieved oocytes	10.03 ± 4.31	4.34 ± 2.82	< 0.001
N0. of MII oocytes	8.42 ± 3.78	2.55 ± 1.10	< 0.001
No. of embryos transferred	1.86 ± 0.35	1.91 ± 0.28	
1	368 (10.9%)	71 (39.7%)	< 0.001
2	3019 (89.1%)	108 (60.3%)	

Data are presented as Mean ± SD or n/total (%). BMI: body mass index; FSH: follicular stimulation hormone; E2: estradiol; P: progesterone; LH: luteinizing hormone; AFC: antral follicle count; Gn: gonadotropin; HCG: human chorionic gonadotropin

### Pregnancy outcomes

Comparison of pregnancy outcomes between the two groups in Table 2. No significant difference in embryonic development parameters such as cleavage rate, and undesirable pregnancy outcomes such as ectopic pregnancy rate, pregnancy loss rate. There was statistically significant difference in the implantation rate (33.2% vs. 18.5%, P < 0.001), clinical pregnancy rate (50.4% vs. 27.4%, P < 0.001), live birth rate (36.3% vs. 20.1%, P = 0.036) (Table 2).

**Table 2:** Comparison of pregnancy outcomes between the two groups

	group 1 ≤ 0.367(n = 3387)	group 2 > 0.367(n = 179)	P
MII maturity rate (%)	28,530/33,290 (85.7)	456/725 (62.9)	< 0.001
cleavage rate (%)	12,812/13,322 (96.2)	252/264 (95.5)	0.548
ICSI Fertilization rate (%)	12,156/17,903 (67.9)	238/322(73.9)	0.022
implantation rate (%)	2229/6713 (33.2)	53/287 (18.5)	< 0.001
clinical pregnancy rate (%)	1706/3387 (50.4)	49/179 (27.4)	< 0.001
live birth rate (%)	1231/3387 (36.3)	36/179 (20.1)	0.036
Ectopic pregnancy rate (%)	290/1706 (17.0)	4/49 (8.2)	0.103
Miscarriage rate (%)	174/1706 (10.2)	6/49 (12.2)	0.642
Incidence of OHSS (%)	122/3387 (3.6)	1/179 (0.6)	0.031

Multivariate stepwise logistic regression analysis further demonstrated that P/MII ≤ 0.367 group had a positive effect on the pregnancy outcomes in women undergoing ICSI cycle. Compared to the P/MII > 0.367 group, the adjusted OR for clinical pregnancy rate and live birth rate in the P/MII ≤ 0.367 group were found to be 2.47 (95% CI: 1.76–3.46, P < 0.001) and 2.19 (95%CI: 1.50–3.19, P < 0.001), respectively, after adjusting for potential confounders including female age, AFC, E2 level on HCG trigger day, dose of Gn, COS protocols, number of oocytes retrieved, day of embryo transfer, the number of embryo transferred. (Table 3).

**Table 3:** Unadjusted and adjusted OR of pregnancy outcomes in the two groups

Outcomes		OR (95% CI)		P for trend
		group 1 ≤ 0.367(n = 3387)	group 2 > 0.367(n = 179)	
clinical pregnancy rate	Unadjusted OR	2.69 (1.93–3.77)	reference	< 0.001
	Adjusted OR	2.47 (1.76–3.46)	reference	< 0.001
live birth rate	Unadjusted OR	2.27 (1.56–3.29)	reference	< 0.001
	Adjusted OR	2.19 (1.53–3.19)	reference	< 0.001

Adjusted OR and 95% CI were based on the multivariate logistic regression model after adjusting for age, AFC, E2 level on HCG trigger day, dose of Gn, COS protocols, number of retrieved, day of embryo transfer, the number of embryo transferred.

### Neonatal outcomes

We further compared the neonatal outcomes of singleton and twin deliveries between the two groups. As shown in Table 4, singleton delivery in P/MII > 0.367 group had a higher the rate of SGA than those in P/MII ≤ 0.367 group (27.5% vs. 13.0%, P = 0.012). Twin deliveries in P/MII > 0.367 group had a higher the rate of LBW and SGA than those conceived in P/MII ≤ 0.367 group (79.2% vs. 46.5%, P = 0.002) and (62.5% vs. 39.0%, P = 0.032), respectively. No significant differences were found between the two groups in terms of body length, Apgar score, the rates of PTB, macrosomia and LGA (P > 0.05) (Table 4).

**Table 4:** Comparison of singleton and twin neonatal outcomes between the two groups

	Singleton				Twins		
	group 1 ≤ 0.367(n = 889)	group 2 > 0.367(n = 40)		P	group 1 ≤ 0.367(n = 652)	group 2 > 0.367(n = 24)	P
Gender							
Male	438 (49.3%)	22 (55.0%)		0.520	310 (47.5%)	11 (45.8%)	1
Female	451 (50.7%)	18 (45.0%)			342 (52.5%)	13 (54.2%)	
PTB	82 (9.2%)	6 (15.0%)		0.262	309 (47.4%)	15 (62.5%)	0.211
Body length (cm)	49.46 ± 3.31	49.20 ± 2.19		0.625	46.95 ± 2.64	49.00 ± 1.41	0.121
LBW	55 (6.2%)	3 (7.5%)		0.424	303 (46.5%)	19 (79.2%)	0.002
Macrosomia	47 (5.3%)	3 (7.5%)		0.470	4 (0.6%)	0 (0.0%)	1.000
SGA	116 (13.0%)	11 (27.5%)		0.012	254 (39.0%)	15 (62.5%)	0.032
LGA	115 (12.9%)	5 (12.5%)		1.000	23 (3.5%)	0 (0.0%)	0.624
Apgar score	9.74 ± 0.70	9.74 ± 0.50		0.987	9.24 ± 0.93	8.75 ± 0.50	0.296

PTB: Preterm birth (< 37 weeks of gestation); LBW: low birth weight (< 2500 g); Macrosomia (> 4000 g); SGA: Small for gestational age; LGA: large for gestational age

Multivariate stepwise logistic regression analysis further demonstrated that P/MII > 0.367 group had a significant effect on the neonatal birth weight. In the singleton delivery, P/MII > 0.367 group had a higher the rate of newborns being SGA, the adjusted OR in the P/MII > 0.367 group were found to be 3.13 (95% CI: 1.47–6.65, P = 0.003). In twin deliveries, P/MII > 0.367 group had a higher the rate of newborns being SGA and LBW, the adjusted OR in the P/MII > 0.367 group were found to be 3.30 (95% CI: 1.24–7.40, P = 0.015) and 4.44 (95% CI: 1.59–11.86, P = 0.004), respectively, after adjusting for potential confounders including female age, AFC, E2 level on HCG trigger day, dose of Gn, COS protocols, number of oocytes retrieved, day of embryo transfer, the number of embryo transferred. These were consistent with the results obtained from the univariate analysis (Table 5).

**Table 5:** Unadjusted and adjusted odds ratios of singleton and twin neonatal outcomes in the two groups

Outcomes	Singleton				Twins			
	Unadjusted OR (95% CI)	P	Adjusted OR (95% CI)	P	Unadjusted OR (95% CI)	P	Adjusted OR (95% CI)	P
LBW	1.22(0.37–4.11)	0.737	1.41(0.40-5.00)	0.593	4.38(1.62–11.86)	0.004	4.44(1.59–11.86)	0.004
SGA	2.53(1.23–5.20)	0.025	3.13(1.47–6.65)	0.003	2.61(1.13–6.06)	0.025	3.30 (1.24–7.40)	0.015

P/MII ≤ 0.367 as reference group. Adjusted ORs and 95% CI were based on the multivariate logistic regression model after adjusting for adjusted for age, AFC, E2 level on HCG trigger day, dose of Gn, COS protocols, number of retrieved, day of embryo transfer,the number of embryo transferred.

## Discussion

The present study analyzed the effects of P/MII ratio on pregnancy and neonatal outcome in 3566 ICSI-ET cycles. Compare to P/MII ≤ 0.367 group, it indicated that the P/MII > 0.367 group had a lower clinical pregnancy rate and live birth rate, furthermore, a significantly higher rate of LBW and SGA were observed in the singleton and twin deliveries. No deleterious impact of high P/MII ratio on embryo quality and undesirable pregnancy outcomes such as ectopic pregnancy rate and pregnancy loss rate was shown.

### Pregnancy outcomes

The study of the impact of P/MII ratio on pregnancy and neonatal outcomes in ICSI cycle is very limited. It was suggested the P/MII ratio higher than 0.32 could impair the endometrial receptivity [27], and P/MII higher than 0.125 could have an unfavorable pregnancy outcomes [22]. However, the effects of the two thresholds value on neonatal outcomes are unclear.

The mechanism of adverse effects of P elevation is a topic of much controversy. Our results indicated that high P/MII ratio did not appear to have a negative effect on cleavage rates, but significantly reduced the clinical pregnancy rate. Therefore, we support this theory: the adverse effect of high P level secreted by multiple follicles during synchronous development on pregnancy, may be impairment of endometrial receptivity rather than on oocyte and embryo quality [28], resulting in embryo–endometrial asynchrony [29], this asynchrony will decrease clinical pregnancy rate.

In our study, the P level on the HCG trigger day in group 2 was higher than that in group 1, but there was no difference in baseline P level between the two groups. This may confirm that ovarian stimulation induces multifollicular development by daily high concentrations of Gn, each follicle will contribute to the P and may lead to total serum P elevation on late follicular phase [30]. Therefore, the high P levels on HCG trigger day can reflect either the normal progesterone secretion of multiple follicles and the high progesterone secretion of a few follicles [20]. Evidence showed that elevated P levels change endometrial gene expression patterns [31] and histological appearance of endometrium [30]. In addition, ovarian stimulation leads to functional change of endometrium after ovulation, reduce the destrogen receptor and P receptor, and a low proliferation index in glands, which may affect the proliferation potential of the endometrium [32]. The aforementioned mechanisms may explain the damage to endometrial receptivity when P elevation, which can impair the implantation potential of embryos.

### Neonatal outcomes

Previous observational studies have suggested that elevated P on HCG trigger day may reduce live birth weight [3], implying that the P on HCG trigger day may be a factor related to fetal growth. Our study has found the effect of P/MII ratio on neonatal outcomes of singletons and twin deliveries to normal ovarian reserve women, and showed that P/MII > 0.367 was independently associated with a risk of SGA in singletons and twin deliveries.

Apart from the fact that SGA is a significant cause of diabetes in adult, it is also related to the increased risk of cardiovascular disease in young Adults [33]. The mainly determined of birthweight of a newborn is by gestational age, therefore, the rates of SGA and LGA are good indicators of neonatal birth weight. However, the exact mechanism of SGA in ICSI cycles has not been clearly elucidated. Aside from intrinsic to the subfertility couple is a risk factor of adverse birth outcomes, the impact multiple births have had adverse effect on birth weight [34, 35]. Furthermore, evidence from human and animal studies suggested that COS leads to histologic changes in the endometrium at the time of implantation [36], it is associated with a reduction in fetal growth [37]. At present, most studies demonstrated that E2 is an independent risk factor of LBW and SGA [4, 38].

One pregnancy [28, 39–41] and perinatal outcomes [42, 43] are ameliorated in the subsequent FET cycle to perform by a “freeze all” policy, because implantation may be improving when the embryo transfer was performed distant from the hormonal changes caused by ovarian stimulation [44]. Our data showed a significant decrease in pregnancy and live birth rates, and a significantly higher rate of LBW and SGA were observed in the singleton and twin deliveries in the P/MII > 0.367 group, therefore, we recommend P/MII > 0.367 to guide the clinical management of patients with high P/MII ratio.

### Strengths and limitations

Our study has several strengths. Firstly, previous studies focused on clinical pregnancy rate and live birth rate, few studies to explore the effect of P/MII ratio on unfavorable neonatal outcomes on fresh cycles. Our study clarified this and provided a theoretical basis of subsequent studies. Secondly, our dataset lies in its large sample and that it represents the singletons and twin deliveries outcomes. The potential weakness of the present study are we sought to focus on normal responders and not on low responders. Whether the unfavorable pregnancy outcomes are due to high P/MII levels or poor ovarian response could be studied more clearly by excluding those women. For this part of the population, we need to carefully select representative cohorts to research the threshold level suitable for them. Then, MII oocytes can only be observed in the ICSI cycle, which limits the generalization ability of the study findings. These findings need to be confirmed in randomized trials to compare differences in pregnancy outcomes between higher and lower P/MII thresholds.

## Conclusion

In summary, P/MII ratio can be a helpful index in ICSI cycles. P level on HCG trigger day as an indicator of endometrial receptivity, and MII oocytes as potential indicators of high-quality mature oocytes in ICSI. We suggest, based on the findings of this research, P/MII > 0.367 would be a more helpful criterion, which can help inform clinical management of patients, considering performed cryopreservation of all embryos. Practically, each ART center should set its own threshold to management women with elevated P concentrations, since the techniques and precision with which the measurement tools and count is performed may led to changes in results. Accordingly, these possible variations may be responsible for the differences in cutoff levels of other research reports.

## Data Availability

The datasets used and/or analysed during the current study available from the corresponding author on reasonable request.

## Abbreviations

P/MII: Serum progesterone on human chorionic gonadotropin trigger day / metaphase II oocyte
ICSI: Intracytoplasmic sperm injection
ET: Embryo transfer
COS: Controlled ovarian stimulation
IVF: In vitro fertilization
CPR: Clinical pregnancy rate
LBR: Live birth rate
PTB: Preterm birth
SGA: Small for gestational age
LGA: Large for gestational age
LBW: Low birth weight
OHSS: Ovarian hyperstimulation syndrome
E2: Estradiol
FSH: Follicular stimulation hormone
LH: Luteinizing hormone
HCG: Human chorionic gonadotropin
OR: Odds ratio
SD: Standard deviation
CI: Confidence interval
BMI: Body mass index
P: Progesterone
AFC: Antral follicle count
Gn: Gonadotropin
